# Novel electron microscopic staining method using traditional dye, hematoxylin

**DOI:** 10.1038/s41598-022-11523-y

**Published:** 2022-05-16

**Authors:** Hiroyuki Sasaki, Hisako Arai, Emi Kikuchi, Hideki Saito, Keiko Seki, Takeshi Matsui

**Affiliations:** 1 Department of Occupational Therapy, School of Rehabilitation, Tokyo Professional University of Health Sciences, Tokyo, Japan; 2 Core Research Facilities, School of Medicine, The Jikei University, Tokyo, Japan; 3 Laboratory for Evolutionary Cell Biology of the Skin, School of Bioscience and Biotechnology, Tokyo University of Technology, Tokyo, Japan

**Keywords:** Transmission electron microscopy, Histology

## Abstract

Uranyl acetate (UA) has been routinely used as a staining solution for ultrathin sections used in biological electron microscopy. As a radioactive nuclear material, UA is subject to strict international regulations. To develop an alternative and easy-to-use staining method for ultrathin sections, we examined various commercial light microscopic dyes. We found that Mayer’s hematoxylin followed by Reynold’s lead citrate solution showed staining results comparable to UA and Reynold’s lead citrate solution, and this method is therefore suggested as a reliable and promising alternative to UA staining.

## Introduction

The electron microscopic staining method for biological specimens using uranyl acetate (UA) was reported in 1958 by Watson^1^. Since then, the double staining method of UA followed by lead solution has been used in electron microscopy facilities worldwide owing to its simplicity and optimal staining results^1–3^. Furthermore, array tomography in electron microscopy (EM) with serial section transmission electron microscopy (TEM) or scanning electron microscopy (SEM), serial block-face imaging SEM, and focused ion beam SEM have recently become increasingly popular for a wide range of applications in many biological science disciplines^4,5^. Array tomography offers more flexibility than serial block-face imaging SEM and focused ion beam SEM, because it preserves all sections^4^. Recent technical advances have enabled us to prepare 300–5000 serial ultrathin sections of specimens, stain them with UA, and acquire images by TEM, resulting in terabytes of data. During this procedure, a large amount of UA is required^4,5^. However, obtaining uranyl compounds has recently become difficult because of strict international regulations^6^. Furthermore, restrictions on their use as well as their availability, storage, and disposal, are also expected to become stricter worldwide, because they are used as nuclear materials for weapons.

Although several UA substitutes have been proposed for staining^7–17^, none of them can effectively replace UA. Therefore, UA is still the best choice as a staining solution for electron microscopic studies in biological research fields.

Here, we establish a novel staining method, using a pre-dyeing agent that is easy to handle, as an alternative to double staining with UA and other heavy metals. We examined various basic staining solutions that are conventionally used in light microscopic methods to identify an alternative reagent, which can stain the conventionally prepared thin and semi-thin sections embedded in epoxy resin (Supplementary Fig. 1; Supplementary information).

## Results and discussions

**Figure 1:**
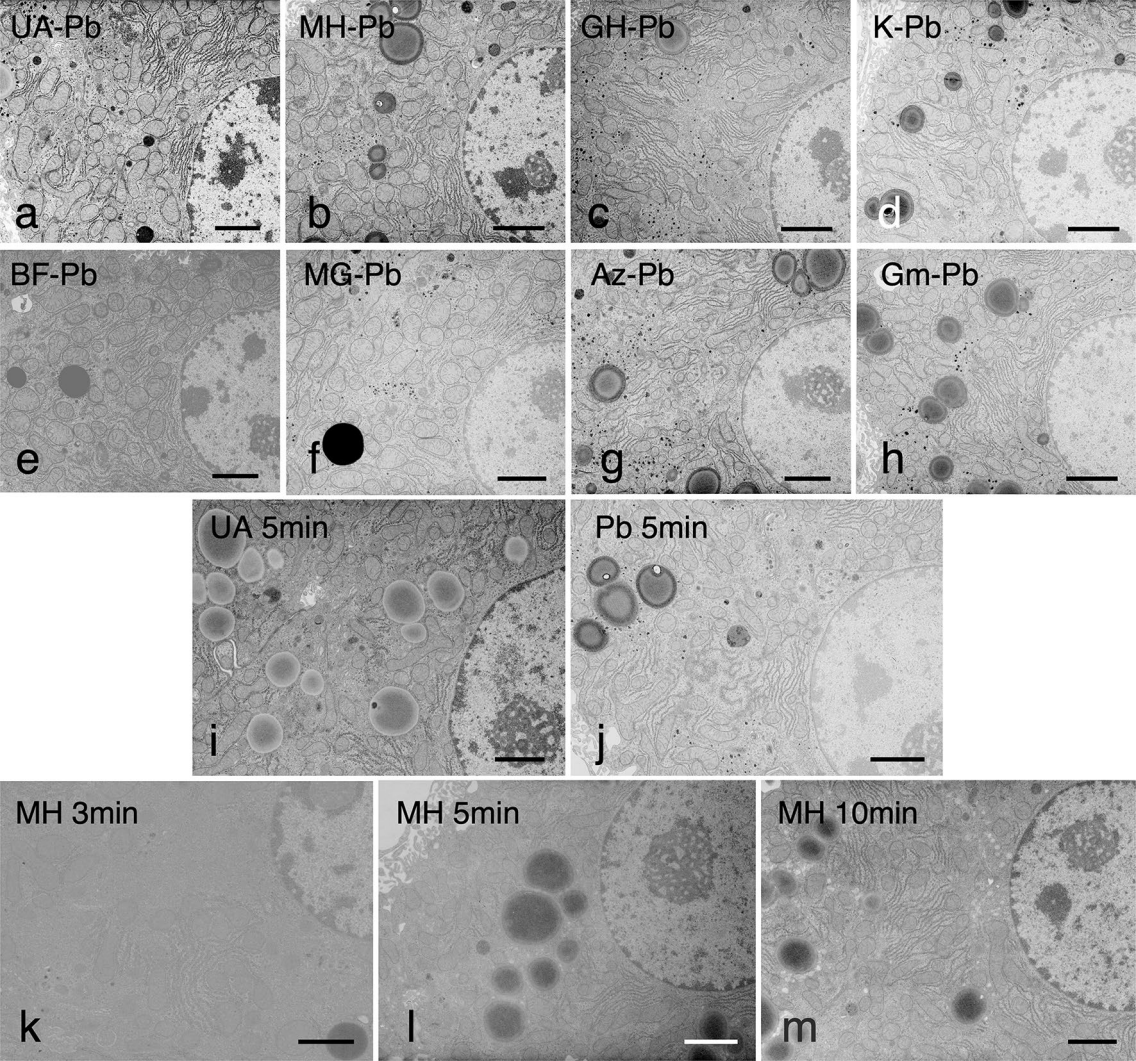
(**a**–**h**) EM images of mouse liver stained with various dyes followed by RPb. Ultrathin sections of mouse liver fixed using the conventional 2% glutaraldehyde and 1% osmium tetroxide fixation and embedded in epoxy resin, doubly stained with (**a**) UA and RPb (UA–Pb), (**b**) MH and RPb (MH–Pb), (**c**) Gill No.3 hematoxylin and RPb (GH–Pb), (**d**) Kernechtrot and RPb (K–Pb), (**e**) basic fuchsin and RPb (BF–Pb), (**f**) methyl green and RPb (MG–Pb), (**g**) alizarin and RPb (Az–Pb), and (**h**) Giemsa solution and RPb (Gm–Pb). Hepatocytes, including the nucleus and cytoplasmic organelles, were stained clearly to different degrees of contrast with all dyes upon post-staining with RPb. The contrast of almost all organelles was highly enhanced upon double staining with MH and RPb, to the same extent as that with UA and RPb. Bar = 2 μm. (**i**—**m**) Ultrathin sections of mouse livers solely stained with (**i**) UA for 5 min, (**j**) RPb for 5 min, (**k**) MH for 3 min, (**l**) MH for 5 min, and (**m**) MH for 10 min. Cells were not clearly displayed in the 3 or 5 min MH single staining, but slight contrast enhancements were observed in the U, RPb and 10 min MH stainings. Bar = 2 μm. The results represent at least three independent experiments with similar results.

Double staining with various dyes (hematoxylins, Kernechtrot, basic fuchsin, methyl green, alizarin red, and Giemsa solution) followed by Reynold’s lead citrate solution^18^ (RPb) (Fig. 1a–h) indicated that the best ultrastructure and image contrast were obtained upon double staining with Mayer's hematoxylin (MH) for 10 min and RPb for 5 min (MH-RPb). The staining effects were generally good, although the image contrast was slightly softer compared to that obtained by double staining with UA for 5 min and RPb (UA-RPb) for 5 min (Fig. 1a). Staining times of 3, 5, 10, 15, and 20 min for MH were attempted and the staining results improved with staining time. The optimal staining time was 10 min because contamination was increased after 15 and 20 min of staining (data not shown). Among various commercial hematoxylin stains (Mayer's, Lilly Mayer’s, Carracci and Gill No.3), MH showed the best contrast (Fig. 1a–h). As shown in Table 1, the hematoxylin content of MH is lower than that of Gill No.3 and Lilly Mayer’s hematoxylins. Furthermore, aluminum ammonium sulfate, chloral hydrate, and citric acid are only present in MH, suggesting that these are responsible for the better electron staining effect. Further investigation will be needed in the future.

**Table 1:** Comparison of components of hematoxylin staining solutions (w/v %).

	Mayer’s hematoxylin	Gill No.3 hematoxylin	Lilly Mayer's hematoxylin	Carracci hematoxylin
Haematoxylin	0.1	0.6	0.5	0.1
Aluminium potassium sulfate	5			5
Aluminium ammonium sulfate			5	
Aluminium sulfate		5.3		
Chloral hydrate	5			
Citric acid	0.1			
Acetic acid			1.2	
Sodium iodate	< 0.1	< 0.1	< 0.1	< 0.1
Ethylene glycol		25		
Glycerine			30	20
Water	< 90	< 70	< 64	< 75

Ultrathin sections of mouse livers were stained solely with UA for 5 min, RPb for 5 min, MH for 3 min, MH for 5 min, or MH for 10 min. Cells were not clearly observed in the 3 min or 5 min MH single staining samples, but slight contrast enhancements of the hepatocytes were observed in the UA, RPb, and 10 min MH stained samples (Fig. 1i–m). Lilly Mayer’s and Carracci hematoxylins showed numerous section breakages, presumably because these stains contain glycerin (Table 1). With the Gill No. 3 stain, there was no structure-specific increase in contrast, and the entire section was stained darkly.

**Figure 2:**
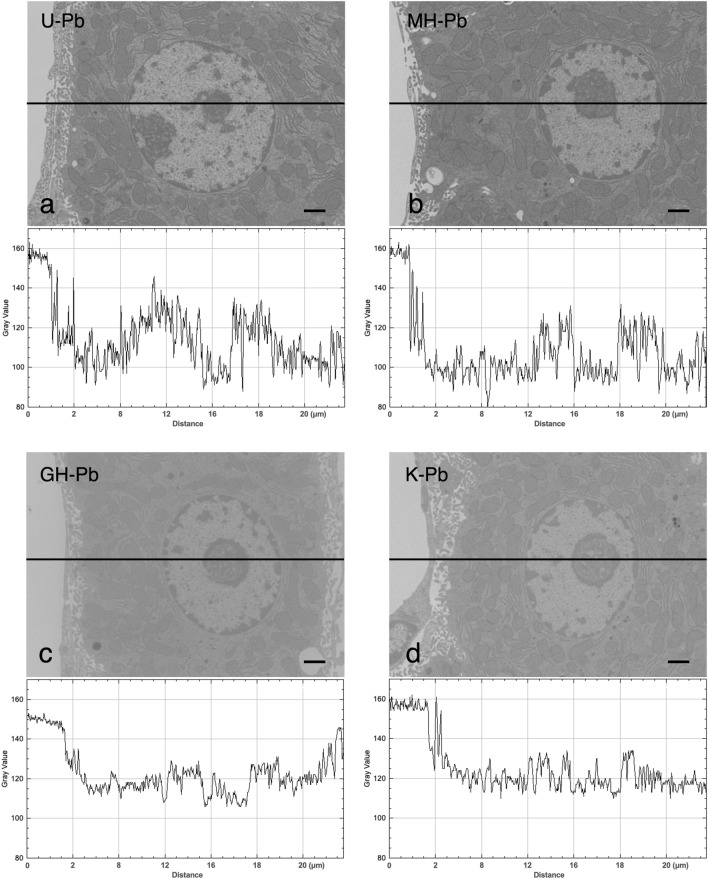
Quantitative analysis of mouse hepatocytes stained with UA, MH, Gill No.3 and Kernechtrot followed by RPb. Digital images were subjected to gray-level (i.e. contrast) line analysis using ImageJ image processing software to semi-quantitatively determine image quality. The line profile displaying two-dimensional graphs of the intensities of pixels along black lines within the images. The x-axis represents distance along the line and the y-axis is the pixel gray-level intensity. The gray level of each pixel is represented by 256 gradations of gray, with pixel value 0 being black and pixel value 255 being white. (**a**) UA-RPb and line profile, (**b**) MH-RPb and line profile, (**c**) Gill No.3-RPb and line profile and (**d**) Kernechtrot-RPb and line profile. Bar = 2 μm.

Digital images were subjected to gray value (i.e. contrast) line analysis using ImageJ image processing software to semi-quantitatively determine the image quality. Figure 2 shows the line profile displaying two-dimensional graphs of the intensities of pixels along black lines within the images. There was a significant difference in contrast between the upper panel (MH-RPb and UA-RPb, Fig. 2a,b) and lower panel (Gill No.3-RPb and Kernechtrot-RPb, Fig. 2c,d). The MH-RPb and UA-RPb had lower gray values (i.e. electron density) compared with Gill No.3-RPb and Kernechtrot-RPb, indicating that MH-RPb and UA-RPb enhanced the contrast relative to the cell membrane systems containing cytoplasmic organelles and nuclei. The MH-RPb and UA-RPb line profiles also exhibited a greater range of gray values in the cells compared with Gill No.3-RPb and Kernechtrot-RPb (approximately 50–60 compared with 20–25, respectively).

**Figure 3:**
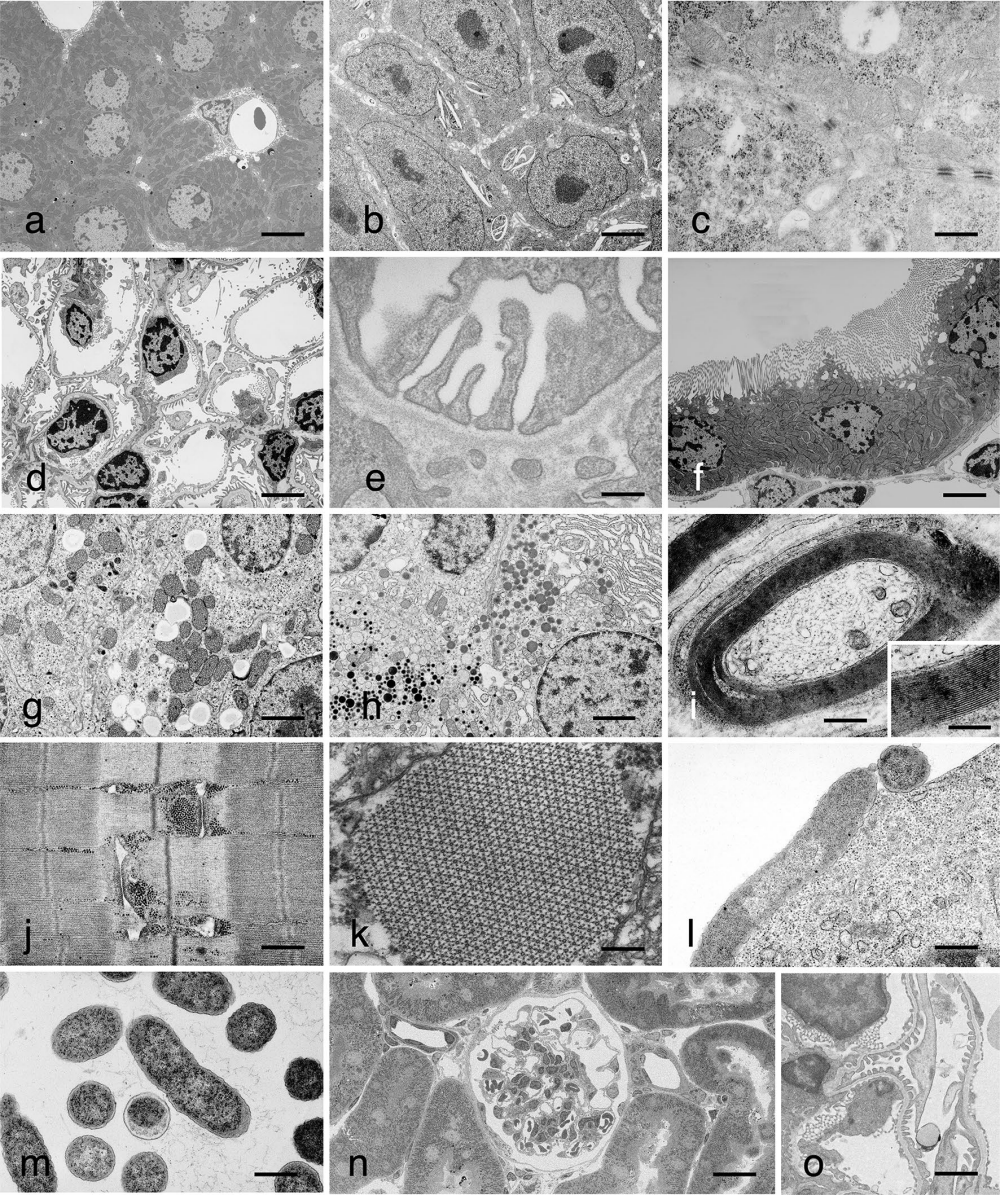
EM images of various kinds of cells and tissues stained with MH followed by RPb. Cells and tissues were prepared using conventional fixation, dehydration, epoxy resin embedding, and sectioning methods. Each section was stained with MH for 10 min followed by RPb for 5 min. (**a**) mouse liver, bar = 5 μm, (**b**) A6 cultured cells, bar = 3 μm, (**c**) A6 cultured cells, bar = 500 nm, (**d**) renal glomerulus, bar = 3 μm, (**e**) renal glomerulus, bar = 500 nm, (**f**) mouse renal proximal tubule, bar = 3 μm, (**g**) mouse adrenal glands, bar = 1 μm, (**h**) mouse anterior pituitary lobe, Bar = 1 μm, (**i**) mouse myelinated nerve, bar = 500 nm, inset shows the high-power view of the myelin region, bar = 200 nm, (**j**) longitudinal section of mouse skeletal muscle, bar = 500 nm, (**k**) cross section of *Drosophila* insect flight muscle, bar = 500 nm, (**l**) *Escherichia coli*-infected Ehrlich tumor cultured cells, bar = 500 nm, and (**m**) *Staphylococcus aureus*, bar = 500 nm. (**n**,**o**) Back scatter image of 200 nm semi-thin sections of mouse kidney observed with FE-SEM: (**n**) shows a low-power view of the renal cortex, bar = 10 μm and (**o**) shows the high-power view of the renal glomerulus, bar = 2 μm. MH is incorporated with little specificity into most of the normally observed tissue components, and although contrast is greatly increased, the general image with certain exceptions is similar to that obtained with UA staining. The results represent at least three independent experiments with similar results.

In MH-RPb, nuclear chromatin was well stained in the liver tissue as well as in various other cells and tissues (Fig. 3). Other organelles such as the cell membrane structures, ribosomes, glycogen, fat droplets, cell adhesion apparatus, and cytoskeletal systems also showed enhanced contrast (Fig. 3a–o). The plasma membrane staining in all samples was also satisfactory. The Z-bands of the skeletal muscle seemed to stain with a lower density, whereas the actin and myosin fibers showed sufficient contrast (Fig. 3j,k). In bacteria, MH-RPb showed the cell walls of *E. coli* and *Staphylococci* with higher affinity and contrast compared to those obtained by double staining with UA-RPb. Additionally, the agar used for bacterial collection also showed staining with high contrast (Fig. 3l–m). Back scatter image of 200-nm semi-thin sections of mouse kidney observed with FE-SEM showed high quality images of the renal cortex (Fig. 3n) and the renal glomerulus (Fig. 3o). Although the entire areas on the slide grass were slightly stained blue, we were unable to observe the structures with a light microscope.

In these all cells and tissues, the roughness of the image quality was unnoticeable, even at a high magnification of approximately 20,000×. Furthermore, good staining results have also been obtained with insect flight muscle embedded in Araldite-506 epoxy resin as another example of a resin compatible for MH staining (Fig. 3k). However, in neural tissue, MH-RPb deposited high electron-dense granules only on the myelin sheath (Fig. 3i inset). The cause of the low-density contrast in the Z-bands of the skeletal muscles are unknown. Electron-dense granular depositions in the myelin sheath may occur in phospholipid-rich structures. Further verification of these phenomena will be needed in the future.

UA and MH have an affinity to macromolecular structures containing nucleic acids, i.e., nucleoplasm and ribosomes^1,19^. However, lead ions of alkaline lead citrate bind to sulfhydryl and carboxyl groups in the tissues, resulting in enhanced contrast by interacting with proteins and glycogens^18^. MH, which was used to prepare tissue specimens in this experiment, was a sufficient alternative to UA. Although single staining of MH and MH-RPb are slightly inferior to that of UA and UA-RPb. The reason for the somewhat inferior single staining effect of MH compared with that of UA and lead may be that the metal it contains, which has electron scattering effects, is aluminum (atomic number = 13), which has a lower atomic number than those of uranium (atomic number = 92) and lead (atomic number = 82). These differences were thought to be compensated for the contrast by the lead used in the post-staining. Of the various kinds of commercial hematoxylin solutions, MH stains nuclei selectively as it contains citric acid. It is a useful staining method that does not require separation by hydrochloric acid in alcohol, or background co-staining^19^. The hematein produced by the oxidation of hematoxylin forms a complex with the aluminum part of the mordant and becomes positively charged. It then binds to the phosphate and/or carboxyl groups of the nucleic acid, which is negatively charged, resulting in staining^19^. It was therefore assumed that the contrast in the EM image was due to the high amount of aluminum-hematein in the MH (Supplementary Fig. 1; Supplementary information).

The uranyl-lead staining procedure dates to 1958. Currently (2022), transmission electron microscopy has evolved into an instrument with greatly improved contrast. Modern electron optics, variable acceleration voltages, variable apertures, high-contrast and high-resolution charge-coupled device (CCD) or complementary metal oxide semiconductor (CMOS) camera image recording, and high-performance image processing software will undoubtedly improve image quality, even for low-contrast specimens. However, it is likely that double staining with uranium acetate and lead will still be used extensively in many electron microscope facilities around the world. MH has advantages, such as a stable supply of commercially and economically available dye solutions and no need to worry about liquid waste (as it is widely used in staining paraffin sections of clinical samples for diagnosis). The staining time is 5–20 min, which is the same as that of UA. However, a disadvantage of MH is that it stains as a dark blue-violet color, which makes it difficult to see the grids during soaking. This can be overcome by staining the grid floating on the droplets of the MH solution.

The International Atomic Energy Agency's "International Basic Safety Standards for Protection against Ionizing Radiation and for the Safety of Radiation Sources" (BSS) has established specific exemption levels, and new regulations for radioactive materials are being developed internationally through legislation^6^. As described above, MH-RPb staining is a simple and useful method compared to staining methods using UA (a radioactive material) in terms of reagent purchase, handling, storage, and liquid waste treatment. It is suitable for large numbers of EM sections in advanced array tomography techniques. Because en bloc staining methods have recently been utilized for TEM and SEM array tomography^4,5^, en bloc staining should be tested for HM in the future.

## Materials and methods

Six-week-old male ICR mouse liver, kidney, anterior pituitary, adrenal gland, skeletal muscle, and peripheral nerve cells. All animal experiments were conducted in accordance with an approved the Institutional Animal Care and Use Committee of The Jikei University School of Medicine protocol. Experiments were conducted in accordance with the National Institute of Health Guide for the Care and Use of Laboratory Animals. Every effort was made to minimize animal suffering. This study is reported in accordance with ARRIVE guidelines (https://arriveguidelines.org). *Xenopus laevis* kidney-derived A6 cultured cells (generously provided by Dr. Yuko Mimori-Kiyosue, Cellular Dynamics Analysis Unit, RIKEN Center for Life Science Technologies) were used as samples for these experiments. *Escherichia coli*-infected Ehrlich tumor cultured cells and *Staphylococcus aureus*^20^ were also used. Furthermore, chemically fixed and Araldite-506 embedded *Drosophila* insect flight muscle samples (generously provided by Dr. Clara Franzini-Armstrong, Department of Cell and Developmental Biology, University of Pennsylvania School of Medicine) were used to confirm the staining effects.

Each sample was double fixed with 2% glutaraldehyde (Nakarai Tesque, Kyoto, Japan) in 0.1 M phosphate buffer and 1% osmium tetroxide (Nakarai Tesque, Kyoto, Japan) in 0.1 M phosphate buffer. The samples were dehydrated in an ethanol ascending series according to standard methods, replaced with propylene oxide, and then embedded in Epon812 resin (Taab Laboratory Equipment, Aldermaston, England). Ultrathin sections of 80 nm thickness were prepared using an EM UC-7 ultramicrotome (Leica Microsystems, Wetzlar, Germany) and then electronically stained using various staining solutions (Supplementary information). Four types of hematoxylin, including Mayer's hematoxylin (Merck, Darmstadt, Germany), Lilly Mayer's hematoxylin (Muto Pure Chemicals, Tokyo, Japan), Carracci hematoxylin (Muto Pure Chemicals, Tokyo, Japan), and Gill No.3 hematoxylin (Merck, Darmstadt, Germany), as well as other light microscopic dyes including Kernechtrot (Muto Pure Chemicals, Tokyo, Japan), basic fuchsin (Tokyo Chemical Industry, Tokyo, Japan), methyl green (Tokyo Chemical Industry, Tokyo, Japan), alizarin red (Nakarai Tesque, Kyoto, Japan), and Giemsa solution (Merck, Darmstadt, Germany) were used for electron microscopic staining. A saturated UA solution was used as the control (Supplementary information).

The staining solutions were filtered through a Millipore filter (0.45 μm, Merck, Darmstadt, Germany) before use. Staining was performed with each staining solution for 3, 5, 10, 15 and 20 min, respectively, followed by RPb for 5 min. As a control, conventional double staining was performed using saturated UA solution for 5 min and RPb for 5 min. Furthermore, sections were solely stained MH, UA or RPb, respectively.

The samples were observed and recorded using a Hitachi H-7500 transmission electron microscope (Hitachi High Technologies, Tokyo, Japan) with a 20 µm objective lens aperture at an acceleration voltage of 100 kV. The images were digitized with a 1024-pixel (px) × 1024-px AMT Advantage 12HR slow scan CCD camera system (Advanced Microscopy Techniques, Woburn, MA) mounted on a TEM, which was recorded using AMT Image Capture Engine software ver. 5.4.2.40 (Advanced Microscopy Techniques, Woburn, MA). To determine the staining quality, the brightness and contrast was maintained during image acquisition with the AMT Image Capture Engine. The resulting digital images of each image were subjected to gray level (contrast) line analysis using ImageJ (National Institute of Mental Health, Bethesda, Maryland, USA) image processing software to determine the image quality. Additionally, 200-nm sections prepared with an EM UC-7 ultramicrotome (Leica Microsystems) attached to glass slides were stained with hematoxylin for 20 min and with RPb for 5 min, and backscattered electron images were obtained using a Hitachi Regulus 8240 field emission scanning electron microscope attached to an yttrium aluminum garnet (YAG) backscatter electron detector (Hitachi High Technologies, Tokyo, Japan) with 1280-px × 960-px at an acceleration voltage of 5 kV (Supplementary information).

## Supplementary Information

Supplementary Information.

## Data Availability

The datasets generated and/or analysed during the current study available from the corresponding authors on reasonable request.
