# Supplementary material for: Novel electron microscopic staining method using traditional dye, hematoxylin
Source: Sci Rep. 2022 May 16;12:7756. doi: 10.1038/s41598-022-11523-y (PMC9110702; doi:10.1038/s41598-022-11523-y)
Supplement: Supplementary file 1 — Supplementary Information. [file 41598_2022_11523_MOESM1_ESM.docx]

**Supplementary Information** ****

**
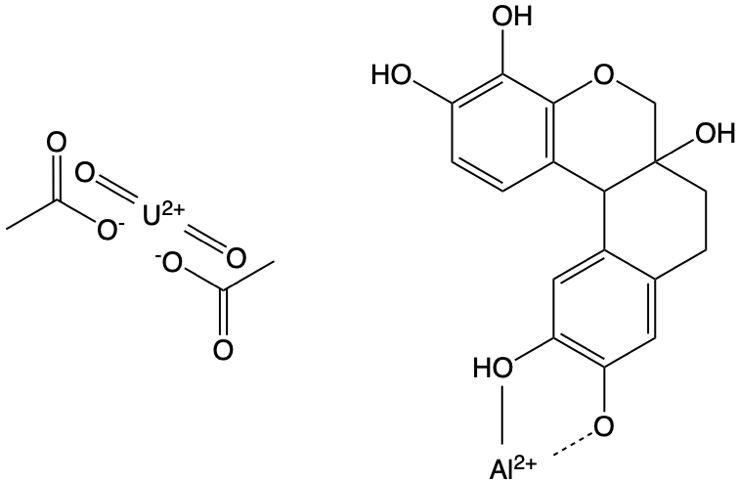
**

**Supplementary figure 1.** **Procedure of sample preparation for EM observation.**

Tissues or cells are doubly fixed with glutaraldehyde and osmium tetroxide, dehydrated with ethanol, and embedded in Epon812. Samples are sectioned and stained with uranyl acetate followed by lead citrate, and observed by EM. For FE-SEM observations, stained sections are coated with osmium. In this study, we report that the uranyl acetate staining process can be replaced by Mayer's hematoxylin staining.

**Procedure for sample preparation**

- **Fixation and embedding**

Pre-fixation: 2% glutaraldehyde (Nakarai Tesque, Kyoto, Japan) in 0.1 M phosphate buffer (pH 7.4) for 3 h at 4 °C.

Rinse: 0.1 M phosphate buffer (pH 7.4) for 10 min, three times at 4 °C.

Post-fixation: 1% osmium tetroxide (Nakarai Tesque, Kyoto, Japan) in 0.1 M phosphate buffer (pH 7.4) for 2 h at 4 °C.

Dehydration: ascending ethanol series, 60%, 70%, and 80% at 4 °C followed by 90%, 95%, and 100% at room temperature (RT) for 10 min each.

Substitution: propylene oxide for 10 min, two times at RT.

Embedding: 1:1 Epon812 epoxy resin (Taab Laboratory Equipment, Aldermaston, England) and propylene oxide overnight at RT, Epon812 for 3 h at RT, and then Epon812 for 2 d at 60 °C.

Thin sections: 80-nm thin sections for transmission EM and 200-nm semi-thin sections for field emission SEM, prepared with a Reichert EM UC-7 ultramicrotome (Leica Microsystems).

- **EM staining and observations**

Pretreatment: Mayer's hematoxylin (Merck, Darmstadt, Germany) was filtered through a Millipore filter (0.45 μm, Merck, Darmstadt, Germany) before use.

Pre-staining: Samples were stained with Mayer's hematoxylin for 10 min for TEM and 20 min for FE-SEM at RT.

Wash: Double-distilled water for 3 min.

Post-staining: Reynold’s lead citrate solution^18^ for 5 min at RT.

Wash: Double-distilled water for 3 min.

Observation and recording of TEM images: Hitachi H-7500 transmission electron microscope (Hitachi High Technologies, Tokyo, Japan) with a 20 μm of objective lens aperture at an acceleration voltage of 100 kV with a 1024-px X 1024-px Advantage 12HR slow scan CCD camera system (Advanced Microscopy Techniques, Woburn, MA) and AMT Image Capture Engine software ver.5.4.2.40 (Advanced Microscopy Techniques, Woburn, MA).

Conductivity coating for FE-SEM: Osmium coating with HPC-1SW osmium coater (Vacuum Device, Ibaragi, Japan)

Observation and recording of FE-SEM images: Hitachi Regulus 8240 field emission scanning electron microscope attached to a YAG backscatter electron detector (Hitachi High Technologies, Tokyo, Japan) with 1280-px X 960-px at an acceleration voltage of 5 kV.
